# Surface Area Determination of Particle-Based Mesoporous Films Using Krypton Physisorption

**DOI:** 10.1021/acsomega.3c09286

**Published:** 2024-01-27

**Authors:** Emma M. Björk

**Affiliations:** Nanostructured Materials, Department of Physics, Chemistry and Biology, Linköping University, 581 83 Linköping, Sweden

## Abstract

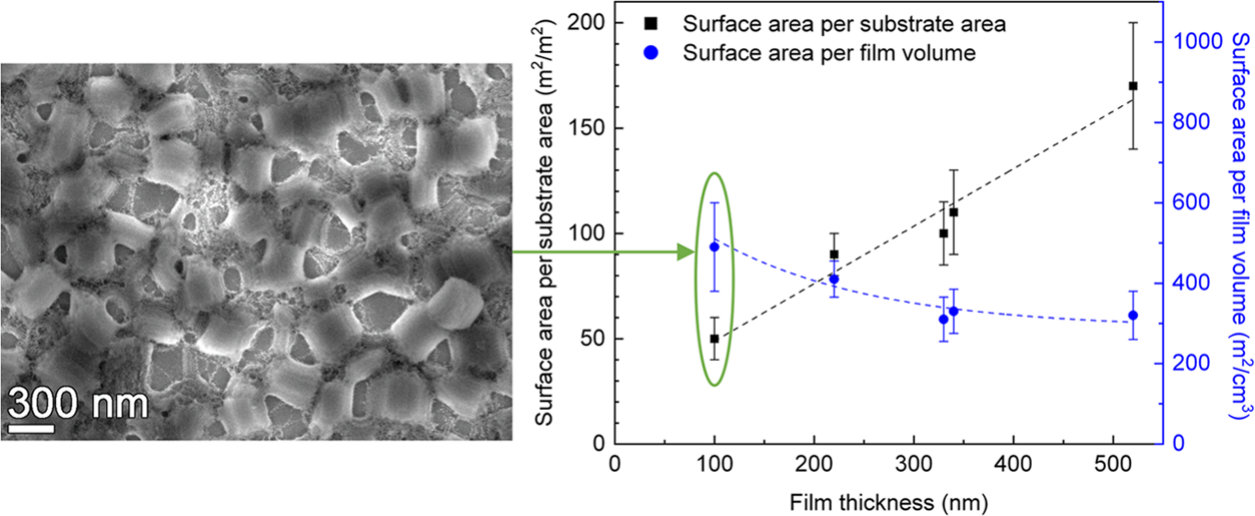

One of the key characteristics of mesoporous films, making them attractive in many applications, is the surface enhancement gained by the porosity. Today, this attribute is rarely presented for particle-based mesoporous films. This work shows that krypton physisorption can be used to determine the available surface area of particle-based silica films synthesized by the direct growth (DiG) method. The surface area per substrate area of the films was measured to be 50–170 m^2^/m^2^ and the surface area per film volume was 310–490 m^2^/cm^3^ when the film thickness was varied between 100 and 520 nm. For comparison, a 105 nm thick film synthesized using dip-coating was used, as this technique renders more smooth films. The corresponding values for the dip-coated film was 90 m^2^/m^2^ and 880 m^2^/cm^3^, showing a higher surface area per film volume for the continuous film. The method is hence suitable for the characterization of mesoporous films synthesized with various techniques and can be used for the optimization of catalysts, drug delivery systems, and sensors.

## 1 Introduction

Mesoporous films are, with their tunable porosity and large surface area, of interest in vital applications such as drug delivery,^1,2^ catalysis,^3,4^ solar cells,^5^ and electronics.^6,7^ In many of these applications, the available surface area of the film is of great importance for its performance and is stated as a key feature, but measuring this characteristic of the film is not a trivial task, and data are rarely presented. For mesoporous powders, nitrogen physisorption is the standard method to use,^8,9^ but this cannot be applied on films as the small amount of film material (<1 mg/cm^2^) on the relatively heavy substrate limits the usage of the technique.^10^ In electrocatalysis, the active surface area of a material can be obtained using cyclic voltammetry, but as this does not correspond to the available surface area of the material, important information regarding, e.g., accessibility of the active sites and transport properties could be lost.^4,11^

Alvarez-Fernandez et al. recently published a tutorial overview of techniques for the structural characterization of mesoporous thin films, where ellipsometric porosimetry (EP) was used for surface area charcterization.^12^ EP allows for the adsorption of various adsorbents on the sample followed by application of t-plot analysis and the Kelvin equation to determine the surface area and pore characteristics of the film.^13^ However, EP requires optical homogeneity and transparency of the films, and the data are fitted to a physical model of the film. As particle-based films have neither of those characteristics and creating a model of such an inhomogeneous system is demanding, EP is not a suitable technique for particle-based films. However, Stassin et al. showed that for thin films of metal–organic frameworks, the surface area information from EP corresponds well with data from krypton physisorption.^14^ Surface acoustic waves (SAWs) can be used to obtain nitrogen sorption isotherms from films.^15^ However, the setup requires that the film is grown on a SAW device, which significantly affects the applicability of the technique.

Krypton physisorption enables specific surface area determination for small sample amounts or materials with a low specific surface area and is a standardized method for surface characterization.^16^ Most often, nitrogen physisorption at 77 K or argon physisorption at 87 K are used for the analysis of specific surface areas, pore sizes, and pore volumes, but these are limited to surfaces above 0.5–1 m^2^. For smaller surfaces, krypton is used as an adsorbate at 77 K. At this temperature, krypton is below its triple point temperature (116 K) and the sublimation pressure is ∼1.6 Torr. For determination of the specific surface area using BET analysis, the saturation pressure of supercooled liquid krypton (2.63 Torr) is used. As the saturation pressure for krypton is approximately 1/300 of that of nitrogen or argon at their respective boiling temperatures, the number of nonadsorbed molecules in the sample tube is significantly reduced, which results in a higher sensitivity of the technique.^8^

Lately, particle-based films have gained interest in drug delivery and catalysis applications. The particle-based films can be synthesized through, e.g., spin-coating particles onto a substrate^17,18^ or the Langmuir–Blodgett methodology.^19^ When the presynthesized particles are used, their characteristics such as pore size and specific surface area can be analyzed using N_2_ sorption. But determining the available surface area of the film on a substrate is difficult due to the limited amount of particles yielding uncertainty in the weight of the film, and potential inhomogeneities in particle deposition. A method for growing particles directly on a substrate, i.e., the DiG method, has been developed by Björk et al.^1,3,20^ The DiG method enables film growth on 3D substrates,^3^ and also film growth on selected areas of a substrate,^1^ which makes them highly interesting as coatings on medical implants. Free particles are a rest product of the synthesis, which also enables determination of pore size and specific surface area here, but the available surface on the film substrate cannot be determined from the particle characteristics.

In this study, a facile method to extract the surface area of various mesoporous films using krypton physisorption at 77 K is investigated. By determining the specific surface area and place in relation to the substrate size, the available surface of the film, which is of great importance for films used in catalytic applications and drug delivery, is obtained. By normalization against the film thickness, it is also possible to compare films with different thicknesses. The method is general and can be applied to films that are synthesized with different methods and chemical compositions.

## 2 Materials and Methods

Hydrochloric acid (≥37%, ACS Reagent), triblock copolymer EO20PO70EO20 (P123) (Mn ∼ 5800, Aldrich), ammonium fluoride (≥98.0%, Fluka), tetraethyl orthosilicate (TEOS) (98%, Aldrich), and heptane (99%, Sigma-Aldrich) were used as received.

### 2.1 Synthesis

Five film-types were synthesized using the DiG method described by Björk et al.^20,3^ Briefly, 2.4 g of P123 and, for some films, NH_4_F (Table S1) were dissolved under stirring in 80 mL of HCl (1.84 M) at 20 °C. 5.5 mL of TEOS and 1 mL of heptane were premixed and added to the solution, and the mixture was stirred for 4 min followed by static conditions overnight at 20 °C. TMCS-treated Si wafers (Supporting Information) were added to the synthesis solution during the static time (Table S1). The films were kept in the synthesis solution during hydrothermal treatment at 100 °C for 24 h. The films were rinsed with distilled water and calcined at 550 °C for 5 h. Finally, the calcined films were cleaned from loose particles by ultrasonication in distilled water for 3 × 5 min. The films are labeled as DiG_X, where X is the film thickness. Specifics regarding the synthesis of different films are presented in Table S1 of the Supporting Information.

### 2.2 Characterization

Kr sorption measurements at 77 K were performed using an ASAP2020 (Micromeritics). The specific surface area was calculated at *P*/*P*_0_ = 0.07–0.20. Three films of each type and thickness were analyzed. The coated area of the substrate was determined as the substrate area (8 × 10 mm^2^) for DiG-synthesized films and estimated using a ruler for the reference dip-coated films. The film thickness of the DiG-synthesized films was determined using cross-sectional SEM with a Leo 1550 Gemini scanning electron microscope (Zeiss) operated at 3 kV. The thickness of the films synthesized with dip-coating was determined using focused ion beam scanning electron microscopy (FIB-SEM) using a Helios Nanolab 600 system operated at 5 kV.^21^ All Kr sorption data are presented in Table S2 of the Supporting Information.

### 2.3 Calculation of the Available Surface

The available surface area per substrate area was calculated as
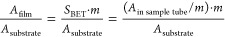
1where *A*_substrate_ is determined using a ruler for dip-coated films and as the cut substrate area for particle-based films, *S*_BET_ is calculated from Kr physisorption on the film on the substrate, and *m* is the sample mass.

## 3 Results and Discussion

**Figure 1:**
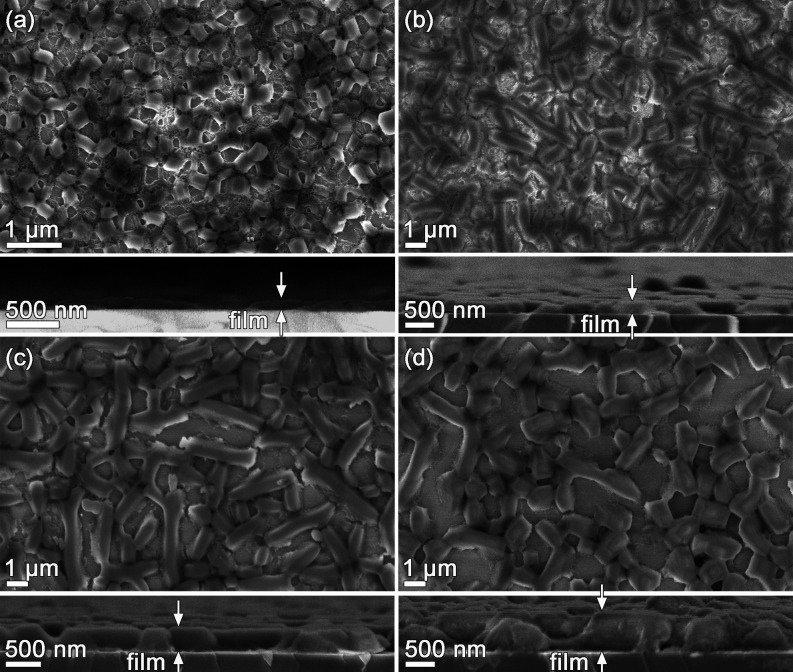
SEM micrographs showing top views and cross-sections of (a) DiG_100, (b) DiG_220, (c) DiG_340, and (d) DiG_520.

The morphologies of films synthesized with the DiG method are shown in Figure 1. The films consist of a monolayer of mesoporous particles directly grown on the substrates. The particles are well separated with cylindrical mesoporous running parallel to the substrate, enabling good diffusion through the pores.^3^ The film thickness was determined from the cross-sectional micrographs, and the data are presented in Table 1. It is clear that NH_4_F and the substrate addition time affect the film thickness. It has previously been shown that NH_4_F has a strong impact on the formation kinetics and particle morphology of both free SBA-15 particles and DiG films, where high concentrations of NH_4_F yield narrow particles that are formed within 10 min, while syntheses with low or no salt concentration produce broad, platelet-shaped particles necessitating several hours to form.^22,23^ The substrate addition time is a narrow window for a successful DiG film growth, as silicated micelles interact with the hydrophobic substrate to act as nuclei for the particle growth.^3^ When free particles are formed in the solution, they cannot grow on the substrate, and nonsilicated micelles will not form cylindrical structures on the substrates for a continued particle growth. The tissue phase between the particles on the substrate is most probable due to slightly silicate micelles forming a mesocellular foam on the substrates.

**Table 1:** Film Characteristics Calculated from Kr Sorption Data

sample	film thickness (nm)	film surface area/substrate area (m^2^/m^2^)	film surface area/film volume (m^2^/cm^3^)
DiG_520	520 ± 60	170 ± 30	320 ± 60
DiG_340	340 ± 20	110 ± 20	330 ± 55
DiG_330	330 ± 30	100 ± 15	310 ± 55
DiG_220	220 ± 20	90 ± 10	410 ± 45
DiG_100	100 ± 10	50 ± 10	490 ± 110
Dip_105	105	90 ± 15	880 ± 130

**Figure 2:**
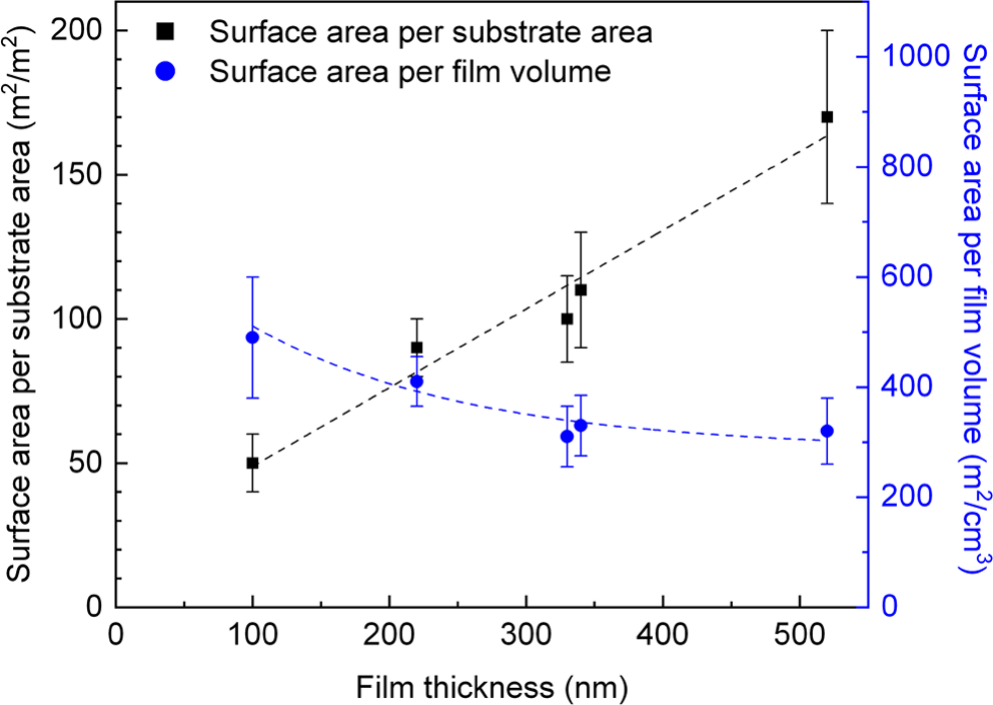
Trends in the available surface area and surface density for DiG-synthesized films.

The calculated film characteristics from the Kr sorption data are presented in Table 1 and Figure 2. At least three films from each batch have been analyzed, and the data are available in Table S2 of the Supporting Information. The available surface area of the film scales well with the film thickness, i.e., the thicker the film, the more surface is available per substrate area, which is expected as larger particles provide more pores. The available surface area per film volume declines with film thickness, from 480 m^2^/cm^3^ for DiG_100 to ∼320 m^2^/cm^3^ for films thicker than 330 nm. The high surface per substrate volume for thinner films can be due to a denser packing of particles, resulting in smaller voids between the particles compared to the thicker films that are formed by larger particles; see Figure 1. In addition, the tissue phase between the particles also contributes to the available surface.

Kr sorption analysis was also performed on a dip-coated film synthesized with F127 as the soft template, resulting in mesopores in the same size range as in the DiG films. All details regarding the film synthesis and SEM micrographs of the film can be found in the Supporting Information. It is apparent that the DiG films have a lower surface area per film volume compared with dip-coated films. Comparing the data for DiG_220 and Dip_105 (Table 1), both films have an available surface area per substrate area of 90 m^2^/m^2^ but the available surface area per film volume is almost half for DiG_220 compared to Dip_105. This is due to the fact that the particle-based films do not have a continuous coverage of the substrate (Figure 1), and the voids between the particles decrease the surface density of the film. The obtained values are in the same range as the surface areas determined using Kr physisorption on other types of mesoporous oxide films. Electrochemically deposited mesoporous ZnO had an available surface per substrate area that varied between 20 and 170 m^2^/m^2^ depending on deposition time,^24^ and for mesoporous TiO_2_ films, it varied between 50 and 70 m^2^/m^2^ depending on substrate functionalization.^25^

The results clearly indicate that Kr sorption is a suitable method for analyzing the surface area of mesoporous films. The method is not limited to films on flat substrates; it is possible to analyze films grown on, e.g., foam or silica films. The measurement is, however, limited to surface area analysis and not pore size distributions due to limitations in recording the full sorption isotherm due to the physical properties of Kr at 77 K and lack of advanced methods required for analyzing the pore size distribution.^14^ Nevertheless, as free mesoporous particles with characteristics identical to those of DiG films are formed during the film synthesis, the pore size distribution can be obtained by regular nitrogen physisorption on the free particles. One practical advantage is that the same instruments often can be used for both nitrogen and krypton physisorption. In this study, we observed the lower limit of the analysis, as the DiG_100 films were not possible to analyze when grown on substrates smaller than 0.8 cm^2^ as it was not possible to obtain good measurements with less than ∼0.0045 m^2^ available surface in the sample tube. This is, however, dependent on the capabilities of the instrument. For small substrates and thin films, it is therefore recommended to measure multiple films simultaneously.

## 4 Conclusions

The study shows that Kr sorption is a suitable technique to determine the surface area of mesoporous particle-based films. By altering the NH_4_F concentration and substrate addition time, it is possible to control the film thickness between 100 and 520 nm of particle-based mesoporous silica films synthesized using the DiG method. The available surface area per substrate unit of these DiG films increases with the film thickness from 50 up to 170 m^2^/m^2^. Comparing DiG films with a dip-coated film with a similar thickness, it is apparent that the dip-coated film has a higher surface area compared to the particle-based film due to its homogeneous structure.

## Supporting Information Available

Methods for substrate functionalization and the dip-coating procedure, table with synthesis conditions for DiG films, extracted data from Kr physisorption isotherms, and SEM micrographs of Dip_105 (PDF)
