# Supplementary material for: Surface Area Determination of Particle-Based Mesoporous Films Using Krypton Physisorption
Source: ACS Omega. 2024 Jan 27;9(5):5899–902. doi: 10.1021/acsomega.3c09286 (PMC10851385; doi:10.1021/acsomega.3c09286)
Supplement: Supplementary file 1 — ao3c09286_si_001.pdf [file ao3c09286_si_001.pdf]

Supporting Information

Surface Area Determination of Particle-Based Mesoporous Films Using Krypton Physisorption

Emma M. Björk

Nanostructured Materials, Department of Physics, Chemistry and Biology, Linköping University, 581 83 Linköping, Sweden

E-mail: emma.bjork@liu.se

Substrate functionalization

Silicon wafers cut to 8 x 10 mm^2 pieces (8 x 15 mm^2 for DiG_100) to be used as substrates. The substrates were cleaned with standard Radio Corporation of America (RCA) cleaning (5 parts H_2O, 1 part H_2O_2 (30 %, Sigma-Aldrich) and 1 part NH_3 (25 %, Emprove Essential) at 85 °C for 10 min) followed by treatment in HNO_3 (≥ 64-66%, Sigma-Aldrich) at ambient temperature for 5 min [32]. The cleaned substrates were rinsed with deionized water and dried in a furnace at 80 °C. Finally, the substrates were exposed to chlorotrimethylsilane (TMCS) fumes for hydrophobic functionalization. TMCS (≥ 99%, Aldrich) droplets were placed around the substrates in the beaker and the beaker was covered with parafilm for 15 min. When the parafilm had been removed, the substrates were exposed to air for at least 5 min prior to adding them to the synthesis solution.

Table S1. Synthesis conditions for DiG films.

**Sample**	**NH_4F amount (mg)**	**Substrate addition time (min)**
DiG_100	28	0.5
DiG_220	7	10
DiG_330	7	12
DiG_340	0	10
DiG_520	0	15

Table S2. Data extracted from Kr physisorption isotherms and SEM cross-sections.

Sample	Sample weight (g)	Substrate area (cm^2)	Specific surface area (m^2/g)	Surface in sample tube (m^2)	Available surface on substrate (m^2/m^2)	Film thickness (nm)	Available surface / film volume (m^2/cm^3)
DiG_100_1	0.1387	1.2	0.0342	0.00474	40	100	395
DiG_100_2	0.1299	1.2	0.0584	0.00759	63	100	632
DiG_100_3	0.1439	1.2	0.0435	0.00626	52	100	522
DiG_100_4	0.1459	1.2	0.0348	0.00508	42	100	423
DiG_220_1	0.0885	0.8	0.0906	0.00802	100	220	456
DiG_220_2	0.0853	0.8	0.0841	0.00717	90	220	408
DiG_220_3	0.095	0.8	0.0679	0.00645	81	220	367
DiG_330_1	0.0974	0.8	0.0851	0.00829	104	330	314
DiG_330_2	0.0899	0.8	0.0737	0.00663	83	330	251
DiG_330_3	0.0947	0.8	0.1013	0.00959	120	330	363
DiG_340_1	0.0978	0.8	0.0946	0.00925	116	340	340
DiG_340_2	0.0956	0.8	0.0772	0.00738	92	340	271
DiG_340_3	0.099	0.8	0.1043	0.01033	129	340	380
DiG_520_1	0.0824	0.8	0.1851	0.01525	191	520	367
DiG_520_2	0.1035	0.8	0.1041	0.01077	135	520	259
DiG_520_3	0.0955	0.8	0.1437	0.01372	172	520	330
Dig_520_4	0.0955	0.8	0.1046	0.00999	125	520	240
Dip_105_1	0.2521	0.98	0.0300	0.00756	77	105	735
Dip_105_2	0.2477	1.12	0.0461	0.01142	102	105	971
Dip_105_3	0.2442	1.16	0.0469	0.01145	99	105	940

Dip coated films

Dip coated films were prepared on silicon wafers decorated with gold nanoparticles as described by Mutschler et al. [1]. Briefly, 36.5 mL ethanol, 1.4 mL water and 37.6 µL HCl (2 M) were mixed in a 100 mL round-bottomed flask. 3.46 mL TEOS a 1.17 g Pluronic® F-127 were added and stirred with 350 rpm at room temperature until the sol became transparent. The sol was aged for 24 h before dip-coating. The dipping was carried out at a relative humidity of about 70 % with a dipping speed of 2.5 mm/s. The films were aged for 30 min at 70% relative humidity before calcination. Calcination was performed in an oven at a temperature of 400 °C for 5 min with a ramp of 20 °C/min.The thickness of films synthesized with dip coating was determined using focused ion beam scanning electron microscopy (FIB-SEM) using a Helios Nanolab 600 operated at 5 kV.

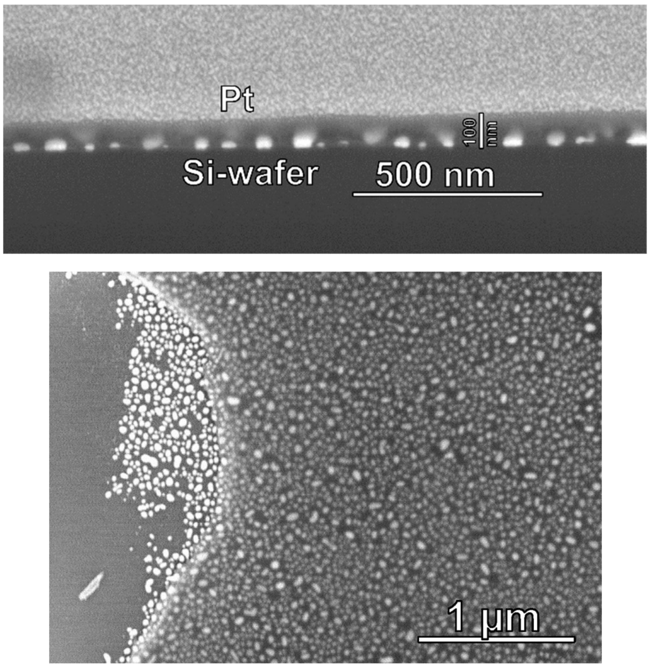

Figure S1. Cross-section and top view SEM micrographs of Dip_105. [1] *Reprinted under Creative Commons Attribution (CC BY) 4.0 license.*[2]

(1) Mutschler, A.; Stock, V.; Ebert, L.; Bjoerk, E. M.; Leopold, K.; Linden, M. Mesoporous Silica-gold Films for Straightforward, Highly Reproducible Monitoring of Mercury Traces in Water. *Nanomaterials* **2019**, *9* (1), 35 10.3390/nano9010035.

(2) http://creativecommons.org/licenses/by/4.0/
